# Role of integrating cannabinoids and the endocannabinoid system in neonatal hypoxic-ischaemic encephalopathy

**DOI:** 10.3389/fnmol.2023.1152167

**Published:** 2023-04-12

**Authors:** Jie Xiao, Yue Zhou, Luqiang Sun, Haichuan Wang

**Affiliations:** ^1^Department of Pathology, Huangshi Central Hospital, Affiliated Hospital of Hubei Polytechnic University, Huangshi, China; ^2^Department of Pharmacy, Xindu District People’s Hospital of Chengdu, Chengdu, China; ^3^Acupuncture and Tuina School, Chengdu University of Traditional Chinese Medicine, Chengdu, China; ^4^Department of Paediatrics, Sichuan Academy of Medical Science and Sichuan Provincial People’s Hospital, School of Medicine, University of Electronic Science and Technology of China, Chengdu, China

**Keywords:** cannabinoids, endocannabinoid system, HIE, neuroprotective, brain development

## Abstract

Neonatal hypoxic-ischaemic events, which can result in long-term neurological impairments or even cell death, are among the most significant causes of brain injury during neurodevelopment. The complexity of neonatal hypoxic-ischaemic pathophysiology and cellular pathways make it difficult to treat brain damage; hence, the development of new neuroprotective medicines is of great interest. Recently, numerous neuroprotective medicines have been developed to treat brain injuries and improve long-term outcomes based on comprehensive knowledge of the mechanisms that underlie neuronal plasticity following hypoxic-ischaemic brain injury. In this context, understanding of the medicinal potential of cannabinoids and the endocannabinoid system has recently increased. The endocannabinoid system plays a vital neuromodulatory role in numerous brain regions, ensuring appropriate control of neuronal activity. Its natural neuroprotection against adult brain injury or acute brain injury also clearly demonstrate the role of endocannabinoid signalling in modulating neuronal activity in the adult brain. The goal of this review is to examine how cannabinoid-derived compounds can be used to treat neonatal hypoxic-ischaemic brain injury and to assess the critical function of the endocannabinoid system and its potential for use as a new neuroprotective treatment for neonatal hypoxic-ischaemic brain injury.

## Introduction

One of the major causes of impairment in newborns is neonatal hypoxic-ischaemic encephalopathy (HIE), which has serious long-term implications for child development (Barata et al., 2019; Zhou et al., 2023). At present, the incidence of perinatal asphyxia ranges between 0.5–1% of all live births, and substantial neurologic damage occurs in as many as 50–75% of these children (Torfs et al., 1990; Ferriero, 2004). Depending on the severity, location, and type of neurologic damage as well as the gestational age, impairments may include a variety of sensorimotor and cognitive abnormalities, which arise at various stages of development and have a considerable effect on children, their families, and society (Du Plessis and Volpe, 2002; Carli et al., 2004). Although neuroprotective treatment has improved, the development of neurological damage remains a substantial issue in HIE cases (Berger and Garnier, 2000).

Currently, neuroprotective measures, such as the rapid identification of affected neonates to enable the timely initiation of therapy, improved monitoring during the perinatal period, strict control during intensive care, and therapies that lessen the developing injury, are urgently needed to minimize the neurological effects of hypoxic-ischaemic brain damage (Shalak et al., 2003; Sanders et al., 2010). For instance, it is important to concentrate on the period directly after the hypoxic-ischaemic episode in neonatal insults because this is when therapeutic approaches can be effective in preventing brain damage. This time frame is typically brief and might range from 2 to 6 h. Therefore, rapid identification would enable easier application of various rescue treatments. Recent studies on neonates have revealed that hypothermia provides varying degrees of neuroprotection, either by preventing DNA breakage and apoptotic cell death after hypoxia-ischaemia (Esteve et al., 1999; Adachi et al., 2001) or by delaying the accumulation of intracellular calcium, decreasing the synthesis of nitric oxide, and decreasing the glutamate concentration in the synaptic space (Hashimoto et al., 2003; Zhu et al., 2004). The only currently available treatment for hypoxic-ischaemic injury in newborns is therapeutic hypothermia, which, despite advancements in its administration, is ineffective in approximately 50% of treated infants (Natarajan et al., 2016). In addition, this treatment has variable efficacy in asphyxiated children and is more effective in treating larger babies than smaller babies (Wyatt et al., 2007). Thus, the complicated pathophysiology of HIE makes treatment challenging and necessitates the development of multiple approaches (Juul and Ferriero, 2014).

Currently, alternative treatments focus on reducing brain damage caused by free radicals by using antioxidant compounds, such as allopurinol, which blocks xanthine oxidase (Palmer et al., 1993; Van Bel et al., 1998) and N-acetylcysteine activity, which reduces apoptosis and inflammation while increasing the intracellular level of glutathione to sequester free radicals (Jatana et al., 2006; Lee et al., 2008). Erythropoietin, which has antiapoptotic and angiogenic effects, is another frequently utilized antioxidant-related medication (Sola et al., 2005) and has been shown to promote neurogenesis and have neuroprotective effects in newborn rats (Chang et al., 2005; Gonzalez et al., 2007). Similarly, melatonin prevents brain damage and the subsequent development of sequelae (Carloni et al., 2008; Signorini et al., 2009). Additionally, substances with anti-inflammatory qualities have been investigated. These include second generation tetracyclines, which prevent microglia from being activated, approaches that increase the lifespan of neurons (Arvin et al., 2002; Jantzie et al., 2005), and statins, which reduce the expression of interleukin-1β and intercellular adhesion molecule 1 (Carloni et al., 2006, 2009). Due to the intricacy of neonatal hypoxic-ischaemic pathophysiology, there is presently no treatment specifically for perinatal brain injuries.

Recent research suggests that cannabinoids are highly effective neuroprotective agents in both acute neurodegenerative conditions, such as hypoxic-ischaemic encephalopathy or traumatic brain injury, and chronic conditions, such as multiple sclerosis (MS), Parkinson’s disease, and Alzheimer’s disease (AD) (Ben Amar, 2006; Maresz et al., 2007). Additionally, cannabinoids have anti-excitotoxic (Marsicano et al., 2003), anti-inflammatory (Chang et al., 2001), and vasodilatory effects (Parmentier-Batteur et al., 2002) and can regulate calcium homeostasis (Barha et al., 2011). Due to their ability to alter glial and neuronal responses, these chemicals have become recognized as neuroprotectants. According to recent research, several anti-inflammatory medications may enhance healing by encouraging neurogenesis after brain injury (Whitney et al., 2009). Because of its anti-inflammatory properties, cannabinoid receptor activation is an important neuroprotective therapy for neonatal hypoxic-ischaemic brain injury (Fernandez-Lopez et al., 2010). In this report, we concentrate on the function of cannabinoids and endocannabinoids and their potential to prevent brain damage caused by neonatal hypoxia and ischaemia.

## Cannabis and cannabinoids

In the 1960s, as marijuana use for recreational purposes increased, anecdotal reports suggested that cannabis could help people with Tourette syndrome, MS, and epilepsy (Cristino et al., 2019). Cannabis, the most widely used illegal recreational drug in the world, comprises approximately 80 phenolic compounds and terpenes, also known as “cannabinoids” (Izzo et al., 2009). As we know, major efforts have been made to pinpoint the chemical components that give marijuana and other cannabis flower preparations their euphoric, perception-altering, and potentially therapeutic effects (Kerai et al., 2018; Lucas et al., 2018). For instance, cannabinoids originating from plants are commonly referred to as phytocannabinoids, of which 9-tetrahydrocannabinol (THC), the main psychoactive ingredient in cannabis, is the most well-known (Izzo et al., 2009). The phytocannabinoid cannabidiol (CBD), in addition to THC, may have an important role in mediating the impact of cannabis on post-traumatic stress disorder (PTSD). While THC is known to exert effects by directly activating cannabinoid receptors, CBD is known to interact with a variety of neurochemical systems, most notably serotonergic and adenosine signalling, and thus its pharmacology is more complex (Carrier et al., 2006; Izzo et al., 2009; Rock et al., 2012). Since the psychoactive effects of THC limit its therapeutic potential and restrict its use in clinical investigations, CBD is more acceptable for clinical development, even for paediatric populations (Devinsky et al., 2016, 2017).

## The endocannabinoid system

**Figure 1:**
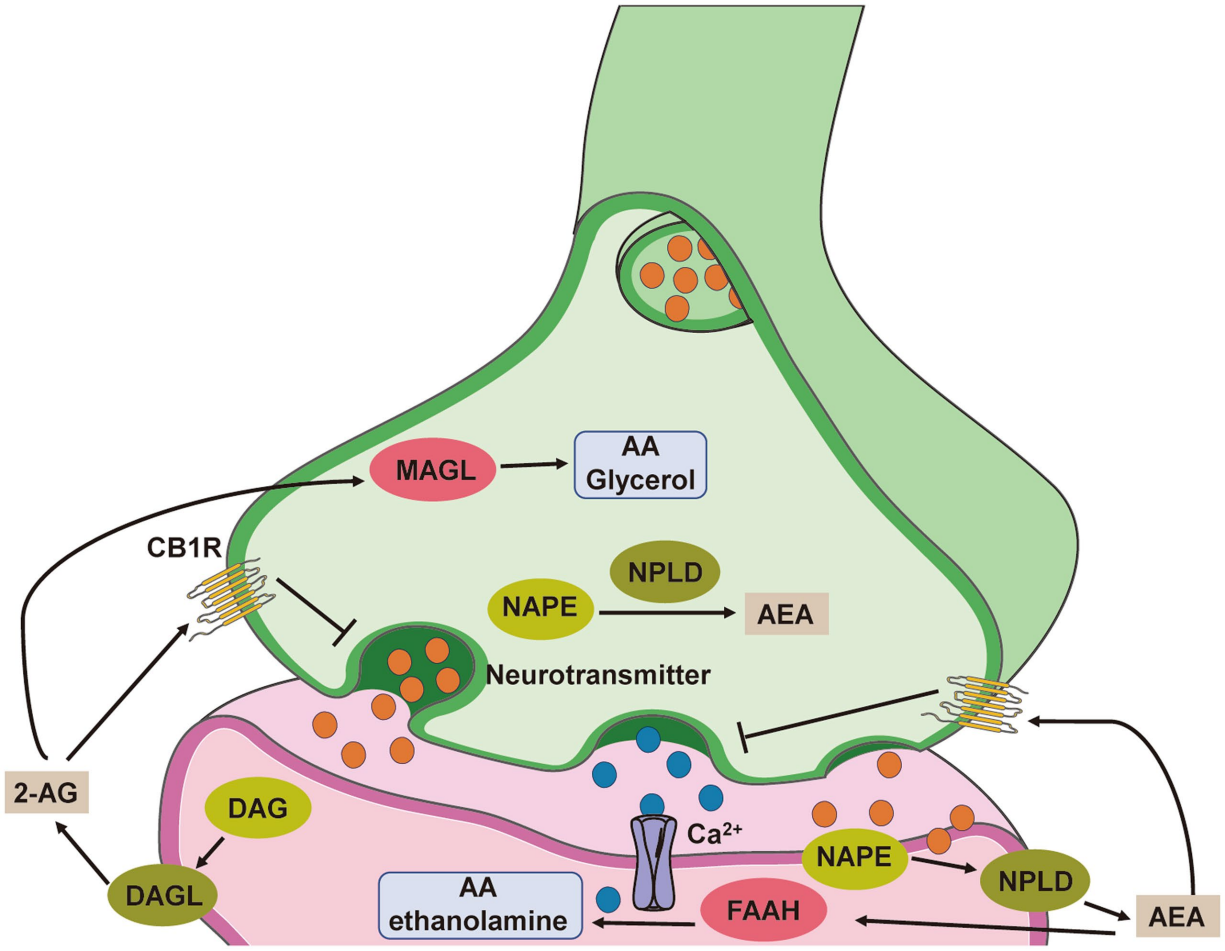
Simplified scheme representing endocannabinoid system-modulated synaptic transmission. The endocannabinoids AEA and 2-AG are not stored in vesicles but instead are synthesized *de novo* from phospholipid precursors through calcium-dependent mechanisms. N-acylphosphatidylethanolamine (NAPE) is hydrolysed by N-arachidonoyl-phosphatidylethanolamine-specific phospholipase D (NPLD) to yield AEA, and diacylglycerol (DAG) is converted to 2-AG by diacylglycerol lipase (DAGL). Both endogenous ligands traverse the synaptic cleft and activate presynaptic CB1 receptors, thereby regulating ion channels and ultimately suppressing neurotransmitter release. Endocannabinoid signalling is terminated following degradation by hydrolytic enzymes in the presynaptic and postsynaptic compartments. Primarily, AEA is converted to arachidonic acid (AA) and ethanolamine by fatty acid amide hydrolase (FAAH) localized to the postsynaptic cell, whereas 2-AG is hydrolysed presynaptically into AA and glycerol by monacylglycerol lipase (MAGL).

Two inhibitory G-protein-coupled receptors (GPCRs), cannabinoid receptor 1 (CB1) and cannabinoid receptor 2 (CB2), as well as two important endogenous ligands, N-arachidonoylethanolamine (anandamide/AEA) and 2-arachidonoylglycerol (2-AG), make up the majority of the endocannabinoid system. Additionally, fatty acid amide hydrolase (FAAH) and monoacylglyceride lipase (MAGL), which hydrolyse AEA and 2-AG, respectively, are metabolic enzymes that considerably influence endocannabinoid signalling (Meyer et al., 2018). The lipid membranes of postsynaptic neurons contain the precursors for AEA and 2-AG. To bind to endocannabinoid receptors in the presynaptic space and control the release of other neurotransmitters, such as glutamate, GABA, dopamine, serotonin, and acetylcholine, AEA and 2-AG are produced as needed and are retrogradely transported across the synaptic cleft (Lovinger, 2008; Jutras-Aswad et al., 2009; Katona and Freund, 2012) (Figure 1).

### Cannabinoid and endocannabinoid neuroprotective mechanism after HIE

Accumulated studies have reported that endocannabinoids modulate the intensity and extent of neurotoxic processes (Barata et al., 2019; Gupta et al., 2020) and the inflammatory response (Chiurchiu et al., 2018; Marinelli et al., 2023) and promote cell survival (Viscomi et al., 2009). Synthetic cannabinoid agonists have shown considerable grey and white matter protection in animal studies of brain injury (Fernández-López et al., 2007). In large animal models of perinatal asphyxia, the cannabinoid WIN55212-2 administered immediately after HI protected against mitochondrial injury and prevented apoptosis (Alonso-Alconada et al., 2010). Cannabidiol given immediately after HI reduced neuronal injury, cerebral haemodynamic impairment, brain oedema and seizures and restored motor and behavioural performance 72 h after HI (Pazos et al., 2013). In rodent models of stroke, prolonged 7-day administration of the cannabinoid WIN55212-2 immediately after injury enhanced neuronal and oligodendrocyte recovery and regeneration in long-term (Fernandez-Lopez et al., 2010). Cannabinoids, however, achieve neuroprotection in part through hypothermia (Leker et al., 2003).

### Endocannabinoid metabolism

In 1992, AEA, the first described endocannabinoid, was discovered in the brain of a pig (Devane et al., 1992). In the mid-1990s, 2-AG, the second most studied endocannabinoid, was found in the intestines of canines (Sugiura et al., 1995). The most prevalent endocannabinoids in the central nervous system (CNS) and all peripheral tissues are AEA and 2-AG. The phospholipid precursors for AEA and 2-AG appear to be produced as needed in the somatodendritic compartment of neurons in response to calcium influx or activation of intracellular phospholipases. Although it is believed that endocannabinoids are instantly produced in response to specific stimuli, there is some evidence that they are transported through cells, stored, and even degraded in adiposomes, suggesting a complicated underlying mechanism of endocannabinoid signalling (Oddi et al., 2008; Kaczocha et al., 2010). 2-AG may be the predominant endogenous agonist of CB2 receptors, and AEA has a higher affinity for CB1 receptors (Pacher et al., 2006). Additionally, it is well known that tissues contain more 2-AG than AEA (Sugiura and Waku, 2002). Although AEA and 2-AG have well-established production and metabolism mechanisms, how these endocannabinoids are transported across the cell membrane remains unknown. Increasing evidence currently points to the possibility that cells can absorb AEA and 2-AG through protein transporter-mediated enhanced diffusion (Yates and Barker, 2009; Nicolussi and Gertsch, 2015).

### Endocannabinoid receptors

The first endogenous CB1 receptor was initially discovered in samples from rat brains (Devane et al., 1988). The cerebral cortex, hippocampus, caudate-putamen, substantia nigra pars reticulata, globus pallidus, entopeduncular nucleus, cerebellum, and spinal cord all have high levels of CB1 receptor expression (Hu and Mackie, 2015). Presynaptic CB1 receptors are primarily found in neurons. Some evidence indicates that only a small percentage of postsynaptic CB1 receptors is found in the mitochondria’s exterior membrane (Benard et al., 2012), where it interferes with the respiratory chain and electron transport, altering brain metabolism and memory formation (Hebert-Chatelain et al., 2016). The CB1 receptors in astrocytes play a role in leptin signalling in the hypothalamus and the modulation of synaptic plasticity in the hippocampus (Bosier et al., 2013; Robin et al., 2018). In addition to stimulating adult progenitor stem cell proliferation and differentiation into neurons or astrocytes, activation of the CB1 receptor has a function in neurodegenerative diseases (Prenderville et al., 2015).

Immune and haematopoietic cells were the first cells to be identified to have the second major endogenous CB2 receptor (Munro et al., 1993; Galiègue et al., 1995). The widespread expression of CB2 receptors in immune cells indicates that endocannabinoids have a unique immunomodulatory function (Lynn and Herkenham, 1994). In addition to traditional immune tissues (thymus, bone marrow, and spleen), other peripheral organs, including the liver (Julien et al., 2005), pancreatic beta cells (Juan-Pico et al., 2006), bone (Ofek et al., 2006), myocardium (Montecucco et al., 2009), and vasculature (Rajesh et al., 2007), express CB2 receptors. According to research on neurological disorders, the main function of the CB2 receptor is immunological regulation. Studies on human brain samples have shown that microglia affected by disorders such as AD, MS, and amyotrophic lateral sclerosis (ALS) have high and specific expression of the CB2 receptor (Aymerich et al., 2018). Furthermore, adult neurogenesis is also stimulated by CB2 receptor activation (Palazuelos et al., 2012), and some data suggest that the CB2 receptor plays a role in controlling the permeability of the blood–brain barrier (BBB) (Chung et al., 2016). According to a study, healthy neurons show very little expression of the CB2 receptor, and CB2 receptor activation produces the opposite effect to that of CB1 receptor stimulation (Navarrete et al., 2013). However, some of these investigations relied on pharmacological or immunological methods that were later discovered to have low selectivity, making the results of these studies questionable (Soethoudt et al., 2017). Finally, it is unclear how CB2 receptors impact neuronal activity. According to one study, functional interaction between the sodium-bicarbonate transporter and the postsynaptic CB2 receptor lowers neuronal excitability in the CA3 and CA2 areas of the hippocampus (Stempel et al., 2016).

## Neurodevelopmental pattern of the cannabinoid and endocannabinoid system

The essential involvement of cannabinoid and endocannabinoid system receptors in important developmental processes, such as neurogenesis, glial formation, neuronal migration, axonal elongation, fasciculation (axonal bundling), synaptogenesis, and synaptic pruning, has been extensively demonstrated in the literature (Berghuis et al., 2007; Mulder et al., 2008; Maccarrone et al., 2014). The major targets of THC are CB1 and CB2 receptors, with the CB1 receptor playing a considerable role in CNS development due to its widespread expression in the developing brain, unlike the CB2 receptor, which has a function that is mostly associated with cells of the microglial/macrophage lineage (Zurolo et al., 2010). In humans, CB1 receptors are present and are functional by the ninth gestational week, which coincides with the start of cortex development. In rodents, CB1 receptors are present and functional from gestational day 11 (Biegon and Kerman, 2001; Zurolo et al., 2010). CB1 receptors are temporarily present on white matter neuronal fibres in both rats and humans during the embryonic stages (Berrendero et al., 1999; Mato et al., 2003). The growth and migration of axons to their final location to establish neuronal pathways may reflect the effects of CB1 receptors on axons or their presence on nonneuronal cells (astrocytes and oligodendrocytes) that direct neuronal migration and axonal elongation. Numerous pluripotent cells carry the CB1 receptor, which controls cell division and proliferation (Maccarrone et al., 2014; De Salas-Quiroga et al., 2015), neural differentiation (Harkany et al., 2007). In postmitotic neurons, CB1 receptor expression and endocannabinoid signalling play crucial roles in the migration and differentiation of glutamatergic and GABAergic cortical cells, cholinergic basal forebrain neurons, GABAergic cerebellar cells, and hypothalamic neurons, according to studies conducted on rodents (Keimpema et al., 2013). Before reaching high levels in early adulthood, when it is ubiquitously expressed and becomes the most abundant GPCR, and the expression of the CB1 receptor is dynamic throughout postnatal development until adolescence (Wang et al., 2003; Mackie, 2005). The adult brain regions with the highest concentrations of CB1 receptors include the cerebral cortex, basal ganglia, hippocampus, and cerebellum (Mackie, 2005), and CB1 receptors are predominantly localized to the synapse on presynaptic terminals (Freund et al., 2003) of both glutamatergic and GABAergic cells (Marsicano and Lutz, 1999).

The two main ligands of the endocannabinoid system, AEA and 2-AG, exhibit divergent ontogenic bioavailability and diverse developmental trajectories. While increasing 2-AG levels throughout embryonic development are correlated with cell differentiation and axonal elongation in the brain, it has been shown that AEA is essential during the early stages of pregnancy for embryo implantation in the uterus (Maccarrone et al., 2014). In addition, 2-AG levels peak at postnatal day 1 and then remain constant until adolescence, when they fluctuate (with high levels during both early and late adolescence) before returning to normal levels in adulthood (Berrendero et al., 1999; Ellgren et al., 2008). In contrast, in the majority of the examined brain areas, AEA concentrations gradually rise from gestational day 21 and peak throughout adolescence (Ellgren et al., 2008; Lee et al., 2013).

## Endocannabinoid signalling in the immature brain and neural cell fate

**Figure 2:**
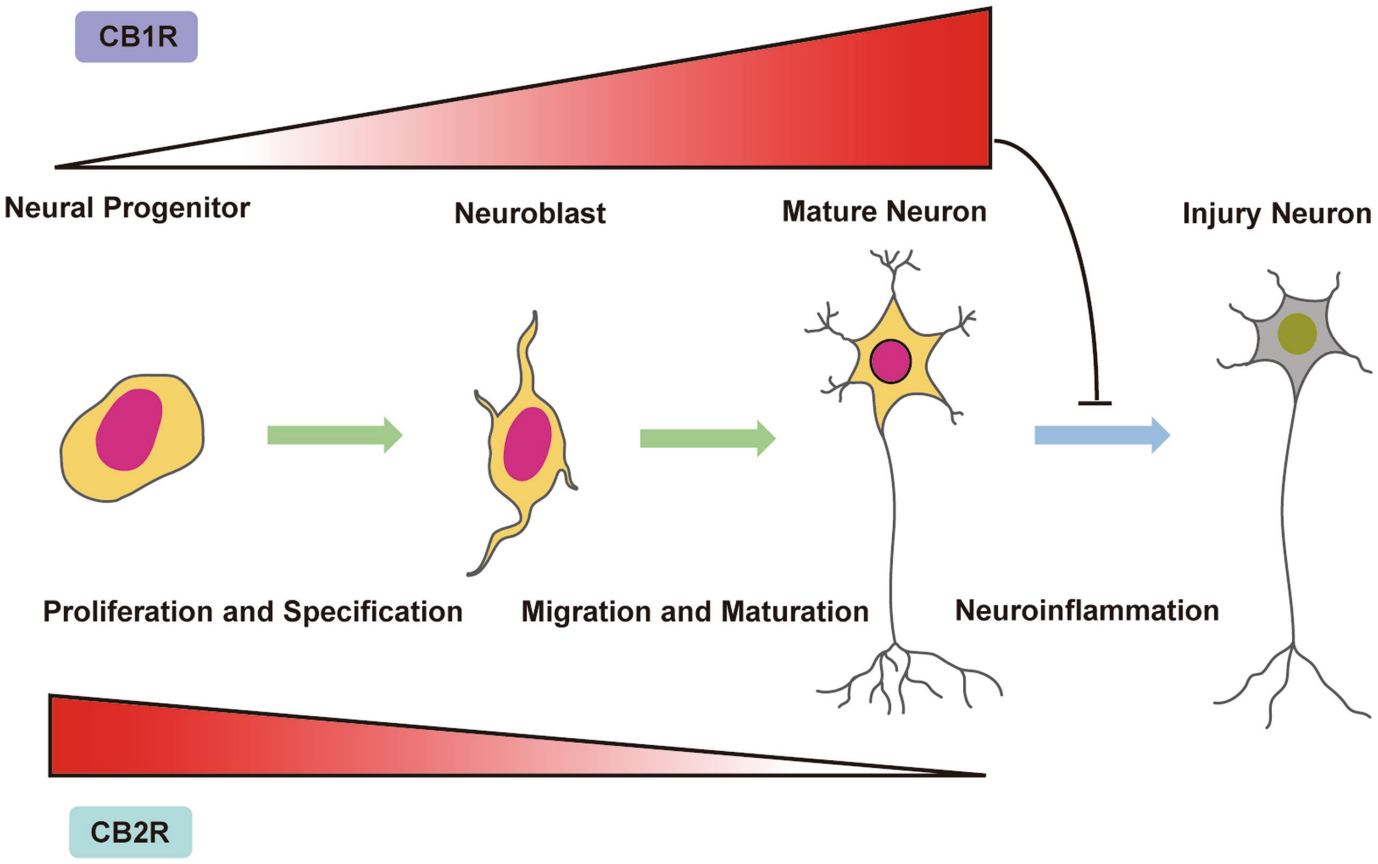
Endocannabinoid system control of neurogenesis and neural cell fate in the immature brain. CB1 receptor expression is present in neural progenitors (NPs) and increases during neuronal proliferation, differentiation and maturation. In contrast, the CB2 receptor is present in NPs and is downregulated upon neuronal proliferation, differentiation and maturation. During neuronal development, CB1 and CB2 receptors control NP proliferation, neuroblast migration and neuron maturation. Under neuroinflammatory conditions, activation of CB1 receptors has been shown to restore adult neurogenesis and decrease the number of injured neurons.

Endocannabinoid signalling effects go well beyond neuromodulation and can even affect the survival of injured neurons. The ability of CB1 and CB2 receptors to communicate across multiple signalling pathways that regulate brain cell formation and maturation during developmental stages is reflected in cannabinoid regulation of neural cell survival (Galve-Roperh et al., 2013; Maccarrone et al., 2014). Therefore, throughout embryonic neurogenesis and during perinatal and adolescent brain development when gliogenesis, myelination and neuron circuit refinement take place, cannabinoid receptors, their downstream signalling pathways and endocannabinoid ligands are all active. Endocannabinoid signalling exerts important cellular plasticity effects that may have an impact on neuronal remodelling of the developing brain in addition to supporting neuronal homeostasis in the adult brain. We next briefly discuss the effects of CB1 and CB2 receptors signalling on neural cell plasticity during brain development (Figure 2).

### CB1 receptor signalling

CB1 receptors are expressed by cells ranging from neural progenitor (NP) cells to fully differentiated neurons with distinctly diverse functions. CB1 receptor signalling in NPs controls cell identity and proliferation, encouraging the shift from radial glial cells to intermediate progenitors (Diaz-Alonso et al., 2015). Later, the ability of CB1 receptor signalling to regulate NP proliferation was found to be conserved in adult neurogenic regions, where CB1 receptors govern the proliferation of hippocampal subgranular cells (Aguado et al., 2007). The CB1 receptor is also active in the subventricular zone and influences oligodendrogenesis and neurogenesis (Xapelli et al., 2013). Hemopressin, a CB1 receptor modulator, has been demonstrated to encourage SVZ-derived oligodendrogenesis in newborn mice (Xapelli et al., 2014). In addition, a study of genetic engineering of FAAH and diacylglycerol lipase (DAGL), the key enzymes responsible for AEA breakdown and 2-AG synthesis, respectively, confirmed that endocannabinoid signalling controls adult neurogenesis in a manner consistent with findings from endocannabinoid receptor knockout mouse models (Gao et al., 2010).

### CB2 receptor signalling

The vast majority of neuronal populations lack the CB2 receptor, and its function in normal physiological brain function is a current topic of study. However, the importance of CB2 receptor signalling has been shown in cases of neurodegenerative diseases and nervous system injury. CB2 receptors are mostly recognized for their capacity to regulate neuroinflammation, and their activation is linked to decreased levels of inflammatory cytokines, innate immunity, and infiltration of peripheral immune cells (Turcotte et al., 2016). Therefore, the CB2 receptor has neuroprotective effects that are primarily due to the regulation of the negative effects of inflammation. Previous studies have reported that inhibition of hippocampal neurogenesis can be prevented by the administration of a CB2 receptor agonist (Avraham et al., 2014); this treatment can also prevent inhibition of oligodendrogenesis in Borna Disease (BD) virus encephalitis (Solbrig et al., 2010). Additionally, notable examples of the positive effects of the CB2 receptor in models of acute inflammation include protection against ageing-related neuroinflammation and reduced neurogenesis (Goncalves et al., 2008; Marchalant et al., 2009). In the APP/PS1 experimental model of AD, CB2 receptor activation can reduce both cognitive decline and hippocampal neurogenesis impairment (Wu et al., 2017).

The CB2 receptor is also expressed in NPs, and in addition to indirect regulation of neurogenesis and neuroprotection, its activity regulates cell proliferation and neurogenesis in a cell-autonomous manner (Palazuelos et al., 2012). As their activity promotes neuroblast migration towards the damaged cortex, CB2 receptors are known to be involved in brain encephalopathies (Bravo-Ferrer et al., 2017). These studies have highlighted the role of endocannabinoid signalling, including that of both the CB1 and CB2 receptors, in neuroblast migration along the rostral migratory stream (Oudin et al., 2011). Overall, the role of endocannabinoid signalling in neuronal development and plasticity is demonstrated by the capacity of the CB1 receptor to connect to numerous signalling pathways involved in neural precursor cell proliferation, neuronal differentiation, and survival. Furthermore, the therapeutic effects of cannabinoids in the treatment of brain encephalopathies and injuries to the developing brain are explained by the complementary effects of CB2 receptor signalling on neural cell survival. Notably, the development of CB2 receptor-specific manipulation techniques can mitigate the negative effects of neuroinflammation without causing the side effects that are associated with typical neuronal CB1 receptor activity.

## Therapeutic potential of the cannabinoid and endocannabinoid system after hypoxia-ischemia

Several studies have proposed the involvement of the cannabinoid and endocannabinoid systems in a variety of activities, including the modulation of calcium homeostasis and excitability, regulation of immune and inflammatory responses (Klein, 2005), activation of cytoprotective signalling pathways (Pacher et al., 2006), and modulation of synaptic plasticity, excitatory glutamatergic transmissions (Freund et al., 2003) and their hypothermic and antioxidant properties (Hampson et al., 2000), although the precise neuroprotective mechanisms of cannabis are not fully understood. In this context, the cannabinoid and endocannabinoid system may additionally serve as a crucial neuroprotective mechanism in both acute and chronic neuronal hypoxic-ischaemic brain injury.

Numerous *in vitro* investigations have documented the neuroprotective properties of cannabis in connection with its antioxidant properties (Marsicano et al., 2002). Cannabis has shown these antioxidant-related neuroprotective effects in *in vivo* models of neurodegenerative disorders (De Lago and Fernández-Ruiz, 2007). Additionally, it has been shown to reduce body temperature (Pertwee et al., 1991). Studies on adult rats using various cannabinoids have shown that a considerable portion of the neuroprotective effect of these substances depends on the presence of hypothermic conditions, as returning the rat body temperature to a normal temperature decreases or even eliminates the positive effect (Leker et al., 2003). Additionally, hypothermia, the current gold standard of treatment is not an easily accessible and 100% curative therapy due to its limited availability and technical complications. There is definitely a need for combination cannabinoid receptor agonist therapies that are easily accessible and have additive neuroprotective effects (Gupta et al., 2020). Previous studies have observed that a single injection of the CB1 synthetic agonist HU-210 significantly reduced body temperature, conferring a strong neuroprotective effect in hypoxic-ischaemic rats, and this beneficial effect was lost when animals were treated with the selective CB1 antagonist SR141716 (Leker et al., 2003). The enhancement of hypothermia by stimulating the endocannabinoid systems or by combined therapies targeting the endocannabinoid system plus hypothermia may have beneficial outcomes in neonates, so these responses are currently under investigation in preclinical models (Lafuente et al., 2016; Barata et al., 2019). Furthermore, cannabinoids cause vasodilation in the brain (Golech et al., 2004), stabilize the BBB and are involved in neuron proliferative processes (Aguado et al., 2006). Cannabinoids improve the energy metabolism of astrocytes (Stella, 2004) and shield these glial cells from cytotoxic and proapoptotic stimuli after brain damage (Docagne et al., 2007).

Previous research has shown that CB1 receptor activation prevents acute stroke through several mechanisms, including the reduction of BBB disruption, a decrease in the volume of infarcted brain tissue, and the induction of hypothermia. These effects are all typically reversed by CB1 receptor antagonists (Chi et al., 2012). Additionally, animals subjected to CB1 receptor deletion have more severe strokes (Parmentier-Batteur et al., 2002), although one study revealed that CB1 receptor antagonists might offer protection in cases of temporary or permanent cerebral artery blockage (Muthian et al., 2004). Similarly, CB2 receptor activation decreases infarct volume and enhances neurological outcome and cerebral microcirculatory function in mice with middle cerebral artery blockage (Zarruk et al., 2012). In fact, palmitoylethanolamide and other N-acylethanolamines protected against transient focal cerebral ischaemia in rats and against the effects of middle cerebral artery occlusion in mice *via* mechanisms that did not require activation of the CB1 receptor but the CB2 receptor or TRPV1 (Franklin et al., 2003). Recent studies have shown that the anti-inflammatory and immunomodulatory effects of cannabis are mediated by CB2 receptors (Fernandez-Ruiz et al., 2007). Numerous studies have demonstrated the anti-inflammatory therapeutic potential of CB2 receptor activation in conditions affecting the central nervous system, including MS, traumatic brain injury, and AD (Mauler et al., 2003; Ni et al., 2004; Ramirez et al., 2005). The presence of CB2 receptors in inflammatory cells in the brain, including microglia (Maresz et al., 2005), has recently been demonstrated, and CB2 receptor expression is induced by hypoxia-ischaemia in the brain (Fernandez-Ruiz et al., 2008). Additionally, CB2 receptor agonists have demonstrated promising results in a variety of neonatal hypoxic-ischaemic brain injury paradigms, reducing cell death and modulating glutamate release, cytokine production, and the expression of cyclooxygenase-2 and iNOS. In an animal model of stroke, it was discovered that the CB2 receptor agonist O-1966 increased blood flow to the brain and reduced neuroinflammation (Sinor et al., 2000). In addition, CB2 receptor activation has been shown to reduce infarct size after middle cerebral artery occlusion and to decrease inflammation-dependent neurodegeneration, reducing the release of inflammatory cytokines and leukocyte adhesion to cerebral vessels (Zhang et al., 2007; Rivers and Ashton, 2010). These findings lend support to the idea that the protective effects of CB2 receptors are primarily due to their anti-inflammatory properties (Castillo et al., 2010). This offers new information on its potential application as a neuroprotective target following neonatal hypoxia.

However, the potential therapeutic effect of CB receptors on ischaemic disorders is far from clear in currently. For example, CB1R activation can promote either protective or toxic responses after brain ischaemia (Pellegrini-Giampietro et al., 2009), as these receptors can either promote the inhibition of glutamate (inducing neuroprotection) or the release of gamma-aminobutyric acid (thus amplifying the toxic response), leading to oxidative stress. In a recent report (Rivers-Auty et al., 2014), the CB2R-selective agonist GW405833 did not show a beneficial effect in a model of cerebral HI, although CB2R-induced neuroprotection has long been known to be related to its anti-inflammatory capacity. Thus, the antioxidant capacity and/or the anti-inflammatory effect developed by the endocannabinoid system after perinatal asphyxia remain a subject of investigation. Further studies should analyse the modulatory effect of CB receptors agonists on ROS and inflammatory cytokine production after HIE, which may contribute to illustrating the role of the cannabinoids and endocannabinoid system in HIE treatment.

Finally, numerous studies have suggested that using synthetic cannabis can lessen damage after brain injury (Fernández-López et al., 2007; Alonso-Alconada et al., 2012; Dai et al., 2014). A histopathological study specifically found that administering WIN55212 soon after recovery from hypoxia-ischaemia successfully reduced brain damage (Fernández-López et al., 2007). Additionally, WIN55212 was shown to prevent the death of apoptotic cells in every area examined by maintaining the integrity and activity of the mitochondria (Fernandez-Lopez et al., 2010) and to encourage neurogenesis in the subventricular zone, oligodendrogenesis, white matter remyelination, and neuroblast production after neonatal hypoxic-ischaemic episodes (Zhang et al., 2009).

## Conclusion

Interest in cannabinoids and endocannabinoids as treatments to manage neonatal hypoxic-ischaemic encephalopathy is supported by the pharmacological characteristics of cannabinoids. In experimental HIE and brain insult models, the administration of cannabis has been shown to have neuroprotective effects. Cannabis preparations may mitigate some of the negative effects of HIE damage in the developing brain. Because cannabinoids have a complicated pharmacology that enables them to target various molecular effectors and receptors, the use of cannabinoid compounds with diverse pharmacological profiles will have distinct effects. Endocannabinoids safeguard the developing brain by inhibiting neuronal excitotoxicity, inflammation, and oxidative stress as well as by altering the fate of neurons and preventing neurodegeneration and harmful glial activation. These cannabis substances provide promising potential clinical applications and raise the possibility of better long-term benefit outcomes for these individuals.

## Author contributions

JX and YZ prepared the first draft of the manuscript. LS collected academic papers and provided critical advice on the manuscript. The study was conceptualized by HW, who also supervised the work and reviewed the entire manuscript. All authors contributed to the article and approved the submitted version.

## Conflict of interest

The authors declare that the research was conducted in the absence of any commercial or financial relationships that could be construed as a potential conflict of interest.

## Publisher’s note

All claims expressed in this article are solely those of the authors and do not necessarily represent those of their affiliated organizations, or those of the publisher, the editors and the reviewers. Any product that may be evaluated in this article, or claim that may be made by its manufacturer, is not guaranteed or endorsed by the publisher.
